# Characterizing the Experience of Tapentadol Nonmedical Use: Mixed Methods Study

**DOI:** 10.2196/16996

**Published:** 2022-06-10

**Authors:** Suzanne K Vosburg, Taryn Dailey-Govoni, Jared Beaumont, Stephen F Butler, Jody L Green

**Affiliations:** 1 Inflexxion, An Uprise Health | IBH Company Irvine, CA United States

**Keywords:** tapentadol, opioid, prescription opioid, nonmedical use, addiction, chronic pain, web-based survey, Bluelight, drug safety

## Abstract

**Background:**

The prevalence of abuse, diversion, and web-based endorsement of tapentadol (extended-release [ER], immediate-release [IR]) has been characterized as low compared with other prescription opioids. Little is known about individual experience with tapentadol nonmedical use (NMU).

**Objective:**

This study aims to pilot web-based survey technologies to investigate the motivation for tapentadol NMU, sources of procurement, routes of administration, tampering methods, doses used, and impressions of tapentadol products (Nucynta and Nucynta ER).

**Methods:**

Recruitment flyers and banner advertisements were placed on the Bluelight website [DragonByte Technologies Ltd] with a link to a web-based survey (Qualtrics) designed to query about individuals’ lifetime tapentadol NMU. This web-based survey was followed by an interactive web-based chat (Cryptocat) with respondents who were willing to be contacted. Respondents were queried about sources for obtaining tapentadol, motives for use, routes of administration, tampering methods, drugs used in combination, tablet strengths and dosages, and reasons for continued or discontinued use. Desirability and attractiveness for NMU was rated.

**Results:**

Web-based recruitment successfully attracted difficult-to-find study participants. A total of 78 participants reported that tapentadol was obtained from friends and family (ER 11/30, 37%; IR 18/67, 27%), the internet (ER 11/30, 37%; IR 12/67, 18%) or participants’ own prescriptions from a doctor (ER 9/30, 30%; IR 17/67, 25%). It was used nonmedically for pain relief (ER 18/30, 60%; IR 33/67, 49%) and multiple psychotropic effects, including relaxation (ER 13/30, 43%; IR 29/67, 43%), reduction in depression or anxiety (ER 7/30, 23%; IR 30/67, 45%), or getting high (ER 12/30, 40%; IR 33/67, 49%). Tapentadol was primarily swallowed (ER 22/30, 73%; IR 55/67, 82%), although snorting (ER 2/30, 7%; IR 8/67, 12%) and injection (ER 2/30, 7%; IR 5/67, 8%) were also reported. The preferred dose for NMU was 100 mg (both ER and IR). The participants reported tapentadol use with benzodiazepines (ER 12/21, 57%; IR 28/47, 60%). Most participants had discontinued tapentadol NMU at the time of survey completion (ER 22/30, 73%; IR 55/67, 82%). Reasons for discontinued ER NMU included side effects (10/22, 46%) and lack of effective treatment (10/22, 46%). Reasons for discontinued IR NMU included lack of access (26/55, 47%) and better NMU options (IR 21/55, 38%). Few individuals were willing to divulge identifying information about themselves for the interactive chat (8/78, 10%), demonstrating the strength of anonymous, web-based surveys. Interactive chat supported the survey findings. A subgroup of participants (4/78, 5%) reported hallucinogenic side effects with high doses.

**Conclusions:**

Web-based surveys can successfully recruit individuals who report drug NMU and those who are difficult to find. Tapentadol NMU appears to occur primarily for pain relief and for its psychotropic effects. Although it was liked by some, tapentadol did not receive a robust pattern of endorsement for NMU.

## Introduction

### Background

The Controlled Substances Act of the United States regulates the use of drugs by placing them into 1 of 5 descending schedules ranging from schedule I (substances with high potential for abuse or dependence and no accepted medical use in the United States) to schedule V (low potential for abuse or dependence and an accepted medical use in the United States). Tapentadol is a centrally acting, atypical analgesic with a novel mechanism of action that is a combination of *μ*-opioid agonist activity and norepinephrine reuptake inhibition used to treat moderate to severe acute and chronic pain [1-3]. It was placed in schedule II by the Drug Enforcement Agency because it was determined to have a high potential for abuse, an accepted medical use in treatment in the United States, and the possibility of leading to severe psychological or physical dependence [4]. Tapentadol immediate-release ([IR]; Nucynta) was approved by the United States Food and Drug Administration in December 2008 and the extended-release (ER) formulation (Nucynta ER) in August 2011 [5]. The abuse liability of tapentadol has been of interest since its release, in part because of its proposed mechanism of action and in part because of its initial identification as a schedule II opioid.

Thus far, the prevalence of tapentadol abuse and diversion (both ER and IR) has been characterized as low compared with other prescription opioid compounds, particularly when considered at the population level [6-8]. For instance, from the fourth quarter of 2011 to the second quarter of 2016, tapentadol had an event rate of 0.015 for intentional abuse, an event rate of 0.029 for diversion, and an event rate of 0.245 for past 30-day use to get high. Comparator opioid active pharmaceutical ingredients (hydrocodone, hydromorphone, morphine, oxycodone, oxymorphone, and tramadol) were reported as being intentionally abused from 7.41 (oxymorphone) to 84.32 (oxycodone) times the rate of tapentadol intentional abuse, diverted from 23.172 (oxymorphone) to 316.862 (oxycodone) times the rate of tapentadol diversion and used for getting high in the last 30 days from 3.48 (tramadol) to 52.97 (oxycodone) times the rate of tapentadol [9]. Tapentadol is associated with the fewest serious adverse events among comparator prescription opioid active pharmaceutical ingredients, as well as the fewest dosage units and prescriptions dispensed in the United States [10]. It has also been associated with a lower risk of seeking out multiple physicians to provide prescriptions than oxycodone [11-13]. Nevertheless, when adjusted for prescription volume or drug availability, low levels of abuse-related outcomes are consistently present [9,14].

### Objectives

It is difficult to make specific inferences about tapentadol abuse or nonmedical use (NMU) because little has been published on user experiences. This is complicated by the comparatively low rates of tapentadol dispensing in the United States [10], low rates of internet posting about tapentadol compared with other prescription opioids [15], and few behavioral pharmacological studies [16]. Therefore, this study sought to address this gap by piloting web-based recruitment for a tapentadol NMU survey.

## Methods

### Overview

The Tapentadol Use Internet Survey (TUIS) is a web-based survey followed by an interactive chat among interested survey completers (Multimedia Appendix 1). The survey queried the motivation for tapentadol NMU, sources of drug procurement, routes of administration, tampering methods, doses used, and impressions of tapentadol products (Nucynta and Nucynta ER). The goal of the survey was to solicit detailed information from individuals who self-reported lifetime tapentadol NMU and to pilot the use of web-based recruitment for the difficulty of finding research participants.

### Definitions

NMU was defined as the use of tapentadol “in a way not prescribed,” including any of the following: (1) used if not prescribed to you; (2) used for reasons other than as a treatment for pain; (3) used via an alternate route of administration (eg, snorted, injected, or other routes not intended for the product); (4) used after tampering (eg, crushed); (5) used in combination with alcohol, illicit drugs, or other prescription drugs without doctor approval; or (6) used at a higher dose than prescribed.

### TUIS Survey

Bluelight.org [DragonByte Technologies Ltd] was selected from a pool of drug discussion websites to host the TUIS because at the time of this study its culture supported authentic posts and harm reduction, the site reported a range of approximately 7000 to 10,000 active users within any 30-day period [17], posts were in English, the site was considered stable because it had been in existence for over 10 years, and the staff encouraged research collaborations. A growing number of studies have included data collected from Bluelight.org [18-23].

The 36-item TUIS was developed to solicit information describing tapentadol NMU. Survey construction was an iterative process whereby investigators designed questions to elicit information about participants’ prescription opioid use history, and, in particular, their experience with and impressions of tapentadol when used nonmedically, the motivation for tapentadol NMU, sources of procurement, routes of administration, tampering methods, doses used, and general impressions of tapentadol products. The survey was reviewed by Bluelight moderators to ensure their research standards were met [24]. After the investigative and Bluelight teams agreed on content, the survey was posted on Bluelight.org.

### Postsurvey Interactive Chat

At the completion of the TUIS, participants were asked to consider chatting interactively with a researcher about their tapentadol product NMU. The chat consisted of 14 questions that provided a framework to enable probing for individual details about initial tapentadol exposure, first use of tapentadol, the high associated with tapentadol, the formulation of tapentadol that had been used for NMU, why it had been used for NMU, number of times using, and the dose of tapentadol most often used for NMU.

### Participants and Inclusion/Exclusion Criteria

Individuals participating in the TUIS had to be at least 18 years of age, able to read and understand the English language, reside in the United States, and be willing to provide consent to participate in the survey. They also had to have visited Bluelight.org and report lifetime NMU of a tapentadol product.

Individuals participating in the postsurvey interactive chat had to have completed the TUIS. They had to be willing to provide contact information (email address or Bluelight username) for chatting purposes. Participants had to have the ability to use a web-based chat program and to provide consent to participate.

### Ethics Approval

The study was approved for conduct by the New England Institutional Review Board (NEIRB 120170005: Internet Survey and Online Chat Interviews Regarding Tapentadol Use).

### Procedures

Participants were recruited for TUIS completion from January through May 2017. A recruitment flyer and banner advertisement were placed on the Bluelight.org website with a link to the web-based survey. This link directed individuals to the consent page which described the voluntary nature of the survey, the absence of payment for survey completion, and information about how to complete the survey. Selecting *I agree to participate* on the informed consent page moved the participant to the beginning of the survey, whereas individuals who did not provide consent to participate in the survey were thanked for their time and brought to an end page. Survey completion took between 5 and 20 minutes depending on the detail of responses provided.

Upon completion of the survey, participants were asked if they would like to be considered for a postsurvey interactive chat regarding their use of tapentadol products. Participants were guaranteed anonymity and asked to provide an email address or a Bluelight username, so they could be contacted by a member of the research team to set up the chat. At the conclusion of the approximately 1-hour, semistructured chat, participants were offered the choice of receiving a US $25 Amazon.com gift certificate or donating the same amount to Erowid.org (a nonprofit drug-education website).

### Web-Based Data Collection and Data Analysis

TUIS responses were collected using the web-based data collection software Qualtrics (Qualtrics) and stored in a secure database. The survey was designed for sequential completion such that it was necessary to complete particular items of interest before moving forward in the survey (forced choice), and responses to previously answered questions were carried forward when requesting more detail. A Qualtrics technology function that blocked more than one survey per individual was enabled. Survey items were examined descriptively with frequency and percentages for categorical and binary variables, and means, medians, error, and ranges for continuous variables. Data analysis was carried out using SAS (version 7.11; SAS Institute).

The postsurvey interactive chat was conducted using Cryptocat, a free, open source, encrypted web-based chat program. Transcripts from the postsurvey interactive chats were saved on secure servers with access permissions given only to research staff for data analysis. Thematic data analysis based on grounded theory qualitative research methodology was used to analyze the interview data [25-27]. This is an analytic approach whereby the raw data drive thematic development and analysis.

Specifically, reviewers conducted in-depth review of the chat content to discern and characterize emerging themes regarding tapentadol NMU. In this open coding phase, 2 reviewers (TDG and JB) read the first half of transcripts and assigned topic categories or themes. Constant comparative analysis was performed by repeatedly going back and reviewing previously established categories. While reviewing transcripts, reviewers continually evaluated whether a certain thought or response from an interview participant fit into a previously existing category or whether it represented a unique new theme. This round of review established a basic coding structure.

Subsequently, the 2 reviewers (TDG and JB) read the remaining transcripts and assigned topic categories or themes as appropriate. If additional categories were discerned, the previous transcripts were revisited to ensure that all categories were adequately captured. In this axial category round of analysis (axial coding uses the predefined concepts and categories while rereading the text to confirm that they accurately represent interview responses and to determine how they are related), reviewers established how the categories related to one another. Finally, final selective coding established overall hypotheses and explanations regarding reasons for, opinions of, and experiences with tapentadol NMU.

## Results

### Internet Survey

#### Participant Characteristics

**Figure 1:**
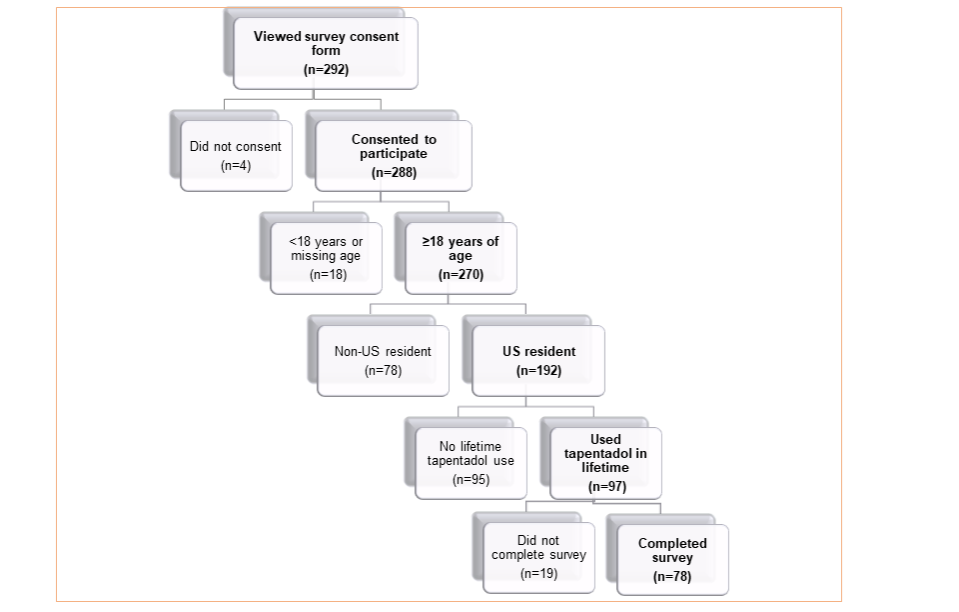
Disposition of survey respondents.

Figure 1 depicts the disposition of the 78 adults who completed the internet survey from January 2017 to May 2017. Those completing the survey were primarily male (67/78, 86%), White (68/78, 87%), aged between 21 and 54 years (21-34 years 46/78, 59%; 35-54 years 17/78, 22%), with a minimum of some college (61/78, 78%). Most opioids (prescription and illicit) were preferred for NMU; however, 58% (45/78) reported a preference for prescription opioids, opiates, or heroin; 13% (10/78) preferred marijuana and cannabis; 8% (6/78) preferred dissociative drugs; 6% (5/78) (each) preferred psychedelics or prescription stimulants; 4% (3/78) preferred benzodiazepines; 3% (2/78) preferred alcohol; and 1 individual (each) preferred 3,4-methylenedioxymethamphetamine or methamphetamine.

A total of 3 participants reported opioid NMU solely with tapentadol products, whereas the remainder of the sample reported lifetime prescription opioid NMU with other opioids in addition to tapentadol. Most began using prescription opioids nonmedically between the ages of 14 and 18 years (44/78, 57%), followed by those aged 19 to 25 years (16/78, 21%), and their first NMU of prescription opioids was hydrocodone IR (29/78, 37%), oxycodone IR (13/78, 17%), or codeine (11/78, 14%). Most patients (45/78, 58%) still used opioids nonmedically.

#### Lifetime Tapentadol NMU

According to the inclusion criteria, all participants reported lifetime NMU of tapentadol: 39% (30/78) reported tapentadol ER NMU, and 86% (67/78) reported tapentadol IR NMU. Almost one-fourth of the sample reported NMU with both ER and IR formulations (19/78, 24%), whereas 14% (11/78) reported NMU solely with tapentadol ER, and 62% (48/78) reported NMU solely with tapentadol IR.

#### Age at First Episode of Tapentadol NMU

Approximately 40% of both tapentadol ER (12/30) and IR (27/67) users reported that they were aged >25 years when they first used tapentadol nonmedically. The remaining ages of the first tapentadol ER NMU were between 14 and 18 (9/30, 30%) years or 19 and 25 (8/30, 27%) years, with an individual aged <10 years. The remaining ages of the first tapentadol IR NMU were between 19 and 25 (25/67, 37%) and 14 and 18 (13/67, 19%) years.

#### Procurement

Table 1 presents the procurement sources of tapentadol for NMU. Tapentadol was often given to participants by family members, friends, or acquaintances (ER 11/30, 37%; IR 18/67, 27%). It was also obtained using the internet (ER 11/30, 37%; IR 12/67, 18%) or from the participants’ own prescriptions from a doctor (ER 9/30, 30%; IR 17/67, 25%). Other sources included being stolen (ER 4/30, 13%), being bought from a dealer (IR 11/67, 16%), or being bought from friends or family (IR 9/67, 13%).

**Table 1:** Sources of tapentadol for nonmedical use organized by formulation.

Source	Tapentadol extended-release (N=30), n (%)	Tapentadol immediate-release (N=67), n (%)
Given to me by a family member, friend, or acquaintance	11 (37)	18 (27)
Internet sources	11 (37)	12 (18)
Own prescription from one doctor	9 (30)	17 (25)
Stolen	4 (13)	6 (9)
Bought from a dealer (someone known to sell drugs)	2 (7)	11 (16)
Bought from a family member, friend, or acquaintance	2 (7)	9 (13)
Own prescription from multiple doctors	1 (3)	1 (2)
Other	1 (3)	3 (5)

#### Motives for Use

Table 2 presents the motives for using tapentadol NMU. Both formulations were mostly used nonmedically for pain relief (ER 18/30, 60%; IR 33/67, 49%) or to feel high, buzzed, or stoned (IR 33/67, 49%). The remaining main motives for NMU included relaxation (ER 13/30, 43%; IR 29/67, 43%) and feeling less depressed or anxious (ER 7/30, 23%; IR 30/67, 45%). Other motives included treatment or prevention of withdrawal symptoms (ER 6/30, 20%; IR 18/67, 27%), treatment of emotional pain (ER 5/30, 17%; IR 26/67, 39%), or feeling more outgoing (ER 6/30, 20%; IR 17/67, 25%) or energetic (ER 6/30, 20%; IR 16/67, 24%).

**Table 2:** Motives for tapentadol nonmedical use organized by formulation.

Motive	Tapentadol extended-release (N=30), n (%)	Tapentadol immediate-release (N=67), n (%)
To provide better pain relief	18 (60)	33 (49)
To relax	13 (43)	29 (43)
To feel high or buzzed or stoned	12 (40)	33 (49)
To feel less depressed or anxious	7 (23)	30 (45)
To feel more outgoing	6 (20)	17 (25)
To feel more energetic	6 (20)	16 (24)
To treat or prevent withdrawal symptoms	6 (20)	18 (27)
To treat emotional pain	5 (17)	26 (39)
To enhance the recreational effects of other drugs or substances	3 (10)	7 (10)
To reduce stress	3 (10)	26 (39)
To ease the comedown from other drugs or substances	2 (7)	7 (10)
To experience psychedelic effects (eg, hallucinations)	1 (3)	3 (5)
Other reason^a^	1 (3)	7 (10)

^a^Tapentadol extended-release other reason: curiosity n=1; tapentadol immediate-release other reasons: curiosity n=5, sexual reasons n=1, chronic Fatigue n=1.

#### Routes of Administration

Table 3 summarizes the routes of administration of NMU tapentadol. Tapentadol was predominantly administered orally, and all patients who swallowed pills reported using alternate oral routes of administration. The participants reported swallowing pills (ER 22/30, 73%; IR 55/67, 82%) or chewing (ER 6/30, 20%; IR 17/67, 25%). Both formulations were ingested using the parachute technique, which refers to wrapping crushed pills in any type of digestible paper (toilet paper or tissue paper) to avoid a bitter taste (ER 6/30, 20%; IR 7/67, 10%). Nonoral routes of administration were used less frequently. Participants reported their preferred route of administration as swallowing whole for each formulation (ER 17/30, 57%; IR 44/67, 66%), followed by chewing (ER 3/30, 10%; IR 7/67, 11%).

**Table 3:** Routes of administration ever used and preferred route of administration for tapentadol organized by formulation.

Route	Tapentadol extended-release (N=30)			Tapentadol immediate-release (N=67)	
	Used, n (%)	Preferred, n (%)	Used, n (%)		Preferred, n (%)
Swallow whole	22 (73)	17 (57)	55 (82)		44 (66)
Chew	6 (20)	3 (10)	17 (25)		7 (11)
Parachute	6 (20)	2 (7)	7 (10)		4 (6)
Drink in solution	3 (10)	2 (7)	4 (6)		1 (2)
Sublingual	2 (7)	1 (3)	6 (9)		3 (5)
Inject	2 (7)	0 (0)	5 (8)		4 (6)
Snort	2 (7)	1 (3)	8 (12)		2 (3)
Other oral	2 (7)	2 (7)	0 (0)		0 (0)
Rectal	2 (7)	2 (7)	5 (8)		2 (3)
Buccal	1 (3)	0 (0)	1 (2)		0 (0)
Smoke	1 (3)	0 (0)	0 (0)		0 (0)

#### Tampering

Table 4 summarizes the tapentadol NMU tampering methods. A subset reported no medication tampering before NMU (ER 9/30, 30%; IR 33/67, 49%). The tampering strategies most frequently used with the ER formulation were breaking into smaller pieces (12/30, 40%), crushing or grinding or shaving (8/30, 27%), chewing (7/30, 23%), or dissolving or soaking (6/30, 20%). Similarly, the most frequently used strategies for IR formulation tampering included crushing or grinding or shaving (16/67, 24%), chewing (15/67, 22%), breaking into smaller pieces (12/67, 18%), and soaking or dissolving (6/67, 9%).

**Table 4:** Tampering methods for nonmedical use (NMU) organized by formulation.

Tampering method	Tapentadol extended-release (N=30), n (%)	Tapentadol immediate-release (N=67), n (%)
No tampering before NMU	9 (30)	33 (49)
Break into smaller pieces	12 (40)	12 (18)
Crush or grind or shave	8 (27)	16 (24)
Chew	7 (23)	15 (22)
Soak or dissolve	6 (20)	6 (9)
Filter dissolved product in liquid using wheel filter, micron filter, or other type of filter	3 (10)	3 (5)
Heat	1 (3)	3 (5)
Cool or freeze	1 (3)	0 (0)
Filter dissolved product in liquid using coffee ball, coffee filter, or other material	0 (0)	3 (5)
Other^a^	0 (0)	1 (2)

^a^Under tapentadol immediate-release, other refers to Dremel tool.

#### Drug Combinations for NMU

The use of tapentadol in combination with other drugs was reported by 70% (21/30) of those who used tapentadol ER for NMU and 70% (47/67) of those who used tapentadol IR for NMU. Table 5 shows that the patterns of drug combinations were similar across both formulations. Benzodiazepines were the most frequently reported drugs used in both formulations (ER 12/21; 57%; IR 28/47, 60%). Alcohol was used at the same rate with ER formulations as benzodiazepines were, but it was used less with IR formulations (IR 18/47, 38%). Other drugs used in combination with tapentadol products were prescription opioids (ER 11/21, 52%; IR 20/47, 43%), marijuana or cannabis (ER 9/21, 43%; IR 17/47, 36%), or prescription stimulants (ER 5/21, 24%; IR 9/47, 19%). Heroin (ER 4/21, 19%; IR 5/47, 11%) and cocaine (ER 2/21, 10%; IR 5/47, 11%) were also taken along with tapentadol.

**Table 5:** Drugs used in combination with tapentadol for nonmedical use^a^.

	Tapentadol extended-release (N=21), n (%)	Tapentadol immediate-release (N=47), n (%)
Benzodiazepines	12 (57)	28 (60)
Alcohol	12 (57)	18 (38)
Rx opioids	11 (52)	20 (43)
Marijuana or cannabis	9 (43)	17 (36)
Rx stimulants	5 (24)	9 (19)
Heroin	4 (19)	5 (11)
Cocaine	2 (10)	5 (11)
Hallucinogens or psychedelics	2 (10)	3 (6)
3,4-methylenedioxymethamphetamine or empathenogenic drugs	1 (5)	2 (4)
Methamphetamine	1 (5)	3 (6)
Antidepressants	1 (5)	5 (11)
Other	0 (0)	4 (9)
Dissociative drugs	0 (0)	0 (0)
Inhalants	0 (0)	2 (4)
Bath salts or 3,4-methylenedioxypyrovalerone	0 (0)	0 (0)

^a^Percentages were calculated with those who reported using drugs in combination with tapentadol.

#### Strength of Tapentadol Tablets Used for NMU and Highest Milligram Amount in One Use Session

Among participants who reported tapentadol ER NMU 57% (17/30) used 100 mg tablets followed by 50 mg and 200 mg strength tablet (33%, 10/30, each). Among participants who reported tapentadol IR NMU, 54% (36/67) used the 100 mg tablet followed by the 75 mg tablet (34%, 23/67) and the 50 mg tablet (31%, 21/67). The pattern of highest milligram amount used in a session was also similar between ER and IR formulations: approximately one-third of ER and IR users reported using between 100 and 200 mg during 1 session (ER 10/30, 33%; IR 24/67, 36%), followed by 251 mg to 500 mg (ER 8/30, 27%; IR 16/67, 24%).

#### End of Use

Most participants with tapentadol ER (22/30, 73%) or IR (55/67, 82%) lifetime NMU no longer used the products at the time of survey completion. Tables 6 and 7 reveal that the primary reasons for discontinuing ER NMU included negative side effects (ER 10/22, 46%), an ineffective high (ER 10/22, 46%), not liking the way the drug felt (ER 8/22, 36%), or ineffective pain relief (ER 8/22, 36%). The primary reasons for discontinued IR use were lack of access (IR 26/55, 47%), availability of better options (IR 21/55, 38%), or an ineffective high (IR 18/55, 33%).

**Table 6:** Reasons for discontinued nonmedical use of tapentadol extended-release.

	Tapentadol extended-release (N=22), n (%)	Tapentadol immediate-release (N=55), n (%)
Have experienced negative side effects	10 (46)	15 (27)
Drug does not provide an effective high	10 (46)	18 (33)
Do not enjoy the way the drug makes me feel (Buzz or Nod)	8 (36)	15 (27)
Drug not effective at pain relief	8 (36)	15 (27)
Better options are available	7 (32)	21 (38)
Tapentadol is too expensive	6 (27)	11 (20)
Do not have access to the drug	6 (27)	26 (47)
Worried about negative side effects	3 (14)	11 (20)
Do not enjoy the psychedelic effects of the drug	2 (9)	4 (7)
Difficult to manipulate product to use via my preferred route of administration	2 (9)	1 (2)
More stigma about this drug than other drugs	1 (5)	3 (6)

**Table 7:** Reasons for continued nonmedical use of tapentadol extended-release and immediate-release.

	Tapentadol extended-release (N=8), n (%)	Tapentadol immediate-release (N=12), n (%)
Drug is effective at pain relief	6 (75)	8 (67)
Enjoy how the drug makes me feel	3 (38)	4 (33)
I have access to the drug	2 (25)	7 (58)
I do not have better options	2 (25)	4 (33)
The drug provides an effective high	2 (25)	3 (25)
Drug is inexpensive	1 (13)	4 (33)
Enjoy psychedelic effects	1 (13)	1 (8)
Not worried about negative effects	1 (13)	2 (17)
Easy to manipulate for my preferred route of administration	1 (13)	1 (8)
Less stigma of this drug	1 (13)	1 (8)
I have not experienced any negative side effects	0 (0)	3 (25)

A small number of participants reported continued NMU of tapentadol ER (8/30, 27%) and IR (12/67, 18%; Table 7). The primary reason for continued NMU in both ER (6/8, 75%) and IR (8/12, 67%) was effective pain relief. The remaining primary reasons were enjoyment of the way the drug felt (ER 3/8, 38%; IR 4/12, 33%) or having access to the drug (ER 2/8, 25%; IR 7/12, 58%).

#### Desirability Ratings of Tapentadol

Survey participants were asked to rate the desirability of opioids they had used nonmedically on a scale ranging from 1 to 100, with 100 representing the best drug imaginable for NMU and 1 representing the worst drug imaginable for NMU. The median values are presented because the number of ratings ranged from 10 (hydromorphone ER) to 67 (tapentadol IR). Table 8 summarizes these findings and illustrates that, compared with other prescription opioid compounds, the median desirability ratings for tapentadol were relatively low (ER=37; IR=41). The highest ratings were for oxymorphone IR (96) and the lowest ratings were for tramadol ER (19).

**Table 8:** Median desirability ratings of opioids used nonmedically.

API^a^	Desirability Rating (Median)	Number of rating and percentage of sample (N=78), n (%)
Oxymorphone IR^b^	96	21 (27)
Oxycodone IR noncombination	90	47 (60)
Oxymorphone ER^c^	86	17 (22)
Hydromorphone IR	85	37 (47)
Morphine IR	84	33 (42)
Oxycodone ER	80	44 (56)
Other	80	16 (21)
Oxycodone IR combination	78	56 (72)
Hydromorphone ER	72	10 (13)
Hydrocodone IR	68	62 (79)
Fentanyl	62	41 (53)
Methadone	61	34 (44)
Morphine ER	60	33 (42)
Hydrocodone ER	53	12 (15)
Meperidine	43	13 (17)
Tapentadol IR	41	67 (86)
Tapentadol ER	37	30 (38)
Codeine	35	49 (63)
Buprenorphine	33	32 (41)
Tramadol IR	24	53 (68)
Tramadol ER	19	22 (28)

^a^API: active pharmaceutical ingredient.

^b^IR: immediate-release.

^c^ER: extended-release.

### Interactive Web-Based Chat

A total of 8 survey participants (10% of the survey sample) agreed to participate in a follow-up semistructured interactive web-based chat. The demographic profile was similar to that of the full sample: 7 (88%) were male, 6 (75%) were White, 6 (88%) were aged <27 years, and 7 (88%) attended a minimum of some college. All participants reported tapentadol IR NMU and half (4/8, 50%) reported tapentadol ER NMU. A total of 6 (75%) participants reported prescription opioids or heroin as their preferred drug for NMU. The remaining 2 (25%) participants reported marijuana and psychedelics (n=1) and dissociative drugs (n=1) as drugs of choice.

Table 9 summarizes the themes and supporting statements derived from participants’ descriptive responses to questions regarding their tapentadol NMU experience. Sentiments and side effects were coded positive if they were favorable or neutral if participants indicated that they neither liked nor disliked the experience or liked one aspect but disliked another. Negative experiences were coded as such if the experience was clearly disliked and typically included extreme or no effects at higher doses.

Of the 8 participants, 3 (38%) reported favorable or positive sentiment, 3 (38%) reported neutral sentiment, and 2 (25%) reported negative sentiment. The side effects of tapentadol were described solely in neutral to negative terms. Reported tampering efforts reflected the composition of the ER formulation (Nucynta ER is formulated with inactive ingredients that make it difficult to crush, although it is not recognized by the Food and Drug Administration as having abuse-deterrent properties): “Nucynta [ER] was like a solid chunk of plastic.” They also reflected the rationale for the oral use of both ER (“I could barely change its shape in the slightest [and] ended up swallowing it whole”) and IR formulations (“If I don’t need to beat a time-release formulation, I typically just swallow 95% of the time”). Comments also reflected the undesirability of the IR formulation for alternate routes of administration: “We actually all tried to snort it… [but] it burned and hurt superbly” (Table 8). Finally, tapentadol was referred to as a fairly obscure find in traditional diversion settings. An individual reported that when trying to sell it or even share it, people “wouldn’t have anything to do with it” because they did not recognize the product.

**Table 9:** Emergent themes from semistructured interview.

Theme		Quotes
**Multiple and varying opinions were expressed about the recreational qualities of tapentadol**		
	Positive opinions	ER was great after I threw up; both ER and IR felt pretty much the same as any other opioid: fuzzy head and body, warm limbs, euphoric... ER [floaty and euphoric; speedy and uplifting], was not as abusable [sic] as IR since it took a long time to peak and lasted so long. IR had intense sedation and euphoria, but [I was] awake during the experience. Strongest opioid feeling I ever felt/Perfect opioid/fell in love with it Liked it but high was unusual—almost stimulating but not really… like maybe half a shot of espresso? ... Hard to say… Very little nausea and histamine reaction, ie, not a lot of itching was a plus, the pills themselves were pretty strong individually and were small [did not have to swallow a bunch of big-ass Percocets, for example]... there was not anything I disliked about it specifically over other opiates…
	Neutral opinions	I cannot say I have any likes or dislikes [about Nucynta ER]: No high whatsoever, slightly more effective than NSAIDs, slightly less effective than even codeine. IR—no high, liked that it helped with withdrawal relief [mild], pain relief [mild]; negative effects are preventive Cannot say it was really pleasant, it’s very overwhelming… Attracted to it for psychedelic potential and novelty of experience; Lower doses helped tremendously with anxiety; Was unique As enjoyable as any other opioid at the equianalgesic dose. Experience was underwhelming, minor mitigation of withdrawal symptoms, similar to how we use codeine
	Negative opinions	Underwhelmed, but kept at it: the higher doses were more rewarding, but the more I used it, the more bad effects I had Dissimilar to other opioids, Costs a lot, no effects, worthless for pain
Tapentadol had side effects described as neutral to negative (5 out of 8 participants)		Auditory hallucinations were neither desirable or undesirable At high dose became extremely visual and extremely disorientating, extremely nauseating. Dislike nausea… Led to hallucinations, no fun Negative effects at high IR dosage would prevent me from using again. Very mild mu opiate with overpowering negative effects [IR felt like kappa agonism: slight dysphoria, slight dissociation] Must stress that when used by itself at too high [a dose] the side effects can be really bad. I spent a couple hours with weird zaps in my head, cloudy thoughts, messed up speech—it was bad and those close to me were worried. It’s anti-abusive nature actually opens up some really crazy effects that were pretty much my worst response to any pain medication
Tapentadol was mostly used orally; tampering efforts were limited to simple methods		Once I learned about soaking it in a carbonated drink for a while, I took the ERs like that I couldn’t crush it [...] the Nucynta [ER] was like a solid chunk of plastic. I could barely change its shape in the slightest. I ended up swallowing it whole Pills swallowed whole/Just took it orally/When it came to pills, if I do not need to beat a time-release formulation, I typically just swallow 95% of the time We actually all tried to snort [insufflate] it [Nucynta IR] through our noses, but we quickly realized that was not an option because it burned and hurt superbly. So, we all took it orally […] I swallowed it whole. It is such a bitter compound that it isn't worth chewing up in my opinion I would cut the [Nucynta ER] pills into quarters with a razor blade and swallow the pieces… it took a little effort
Tapentadol is not well known in traditional diversion settings		It was available [through an] acquaintance who was a pain patient [and] had just had it prescribed. It did not come from a “dealer” per se and I have never seen nor heard of it coming from such a source It’s nowhere on the street, 99% of people have not heard of it and the few times… I tried to share/sell any people would not have anything to do with it because they did not know what it was

## Discussion

### Principal Findings

To date, studies on the abuse liability of tapentadol have focused on aggregate outcome measures [6,7,9,14,15,28]. The present survey sought to address a gap in the tapentadol literature by soliciting direct feedback from individuals with tapentadol NMU experience and characterize the associated motivations, behaviors, and consequences of NMU. To do so, web-based recruitment and survey technology were piloted, and it was found that they were an effective method to recruit a difficult-to-find research sample.

Similar to other prescription opioids [29], the main source of tapentadol was friends, family, or acquaintances. Tapentadol was also obtained on the web and through other sources of diversion, such as being stolen, drug dealers, or purchased directly from friends or family. Although many sources have not reported significant levels of diversion [7,9,14,15], these data reveal a type of tapentadol diversion that is occurring, although perhaps at low levels.

It was hypothesized that individuals might use tapentadol for reasons other than analgesia, such as the rumored psychedelic effects [5,30,31] (Tables 6-8). However, the primary reason for tapentadol NMU and ongoing tapentadol NMU across both formulations was better pain relief, followed by psychotropic effects, including relaxation, reduction in depression or anxiety, or getting high. Approximately 25% to 30% of participants reported that they misused their own tapentadol prescription, revealing NMU among some patients with pain. Pain has been found to contribute to the risk of developing prescription opioid use disorders over time [32], and it is possible that the present data capture aspects of this relationship.

Benzodiazepines are most often used in combination with both formulations of tapentadol. Benzodiazepines can pose a life-threatening risk when used concomitantly with opioids because of the increased risk of respiratory depression and overdose [33,34]. Even so, they are prescribed at varying rates to patients undergoing opioid maintenance therapy. Individuals seeking opiate detoxification also report using benzodiazepines to manage anxiety, help with sleep, decrease opioid withdrawal, enhance the recreational effects of other drugs or substances, or get high [35]. The rationale for the concomitant use of benzodiazepine and tapentadol was not discussed in this study.

Individuals who used tapentadol at high doses (≥200 mg) reported hallucinations. Some interview participants did not identify these effects as positive or negative, but others reported them as strong deterrents to future tapentadol NMU. Hallucinations are described as part of a serotonin syndrome that can occur when taking serotonin and norepinephrine reuptake inhibitor products such as tapentadol [5] and in combination with other serotonergic drugs [36]. To date, the literature is inconclusive as to whether tapentadol alone has resulted in a true serotonin syndrome experience [30,31].

Desirability ratings were lower for tapentadol than for the other opioid compounds. Ratings of desirability were similar between tapentadol ER and IR, with tapentadol ER being less attractive for NMU than IR. In addition to supporting recent findings that IR formulations are more desirable for NMU than ER formulations [37], these data also suggest a lack of desirability for the entire tapentadol molecule, not just for one formulation. In further support of this inference, most participants (61/78; 78%) stopped using either formulation at the time of the survey. These findings were similar to those reported in the study by McNaughton et al [15]. Regardless of how individuals reported using tapentadol, most participants (ER 22/30, 73%; IR 55/67, 82%) did not indicate that they enjoyed it.

### Limitations

Limitations of this study include the use of self-reported data from a self-selected, US-based, convenience sample responding to a pilot internet survey, which may not be a fully representative sample of nonmedical users of tapentadol. The selection of individuals who reported lifetime tapentadol use meant that some did not report their current experiences. The modest number of participants may be because of the market share of tapentadol, but it may also be because of the nondesirability of tapentadol for NMU. This may be a topic for future research [8]. Extension of the recruitment period longer than 5 months might result in a larger sample size, if the reason for the modest sample is the lack of tapentadol market penetration. Notably, few participants volunteered to participate in the follow-up survey. Although it is possible that this activity may not have been of interest, it may also have been because of the requirement to provide an email address or a Bluelight username (which suggests indirect support for anonymous web-based surveys). However, it also suggests an opportunity for another technological development in which survey completers could remain anonymous yet respond to specific follow-up questions. Much of what was reported is similar to other findings in the literature documented herein, lending face value to this report. New directions for future surveys may include patterns of tapentadol NMU, such as frequency of use, redosing, and the degree to which larger doses are used to obtain the same effect. Finally, participants were also aware that the focus of the study was tapentadol and may have felt compelled to over- or underreport its use. Even so, great care was taken to ensure the quality of data collection and analysis.

### Conclusions

In conclusion, these preliminary data reveal potential avenues for further exploration of NMU tapentadol. The use of web-based survey technology for survey recruitment of a difficult-to-find sample and a follow-up interactive chat may be another useful technology for postmarketing surveillance studies. The primary motive for continuing tapentadol NMU was pain relief. Tapentadol ER (12/21, 57%) or IR (28/47, 60%) use together with benzodiazepines were reported . There is also some evidence of diversion. At high doses, psychotropic effects have been reported. Most NMU of tapentadol occurred via oral routes of administration. Similar to other studies, although it was liked by some, tapentadol did not receive a robust pattern of endorsement for NMU.

## Appendix

Multimedia Appendix 1 Tapentadol use internet survey.

## Abbreviations

ER: extended-release
IR: immediate-release
NMU: nonmedical use
TUIS: Tapentadol Use Internet Survey
