# Supplementary material for: Characterizing the Experience of Tapentadol Nonmedical Use: Mixed Methods Study
Source: JMIR Form Res. 2022 Jun 10;6(6):e16996. doi: 10.2196/16996 (PMC9233245; doi:10.2196/16996)
Supplement: Multimedia Appendix 1 [file formative_v6i6e16996_app1.pdf]

Appendix I: Tapentadol Use Internet Survey.

1. Please select your age.

❍ Less than 18 years (1)
❍ Listed ages 18-100; forced choice to select one age.
❍
If Less than 18 years Is Selected, Show: Unfortunately, you are not eligible to complete this survey. Thank you for your interest in participating!

2. What is your gender?

❍ Male (1)
❍ Female (2)
❍ Transgender (3)
❍ Prefer not to answer (4)

3. Where do you live?

❍ United States (1)
❍ Australia (2)
❍ Canada (3)
❍ New Zealand (4)
❍ United Kingdom (5)
❍ Other (Please specify) (6) ____________________

If United States Is Not Selected, Show: Unfortunately, you are not eligible to complete this survey. Thank you for your interest in participating!

4. Please indicate the highest level of education you have completed:

❍ High school / GED or less (1)
❍ Some college (2)
❍ 2-year college degree (Associates) (3)
❍ 4-year college degree (BA, BS) (4)
❍ Master's Degree (5)
❍ Doctoral / Professional degree (MD, JD, PhD, EdD) (6)
❍ Prefer not to answer (7)

5. How would you best describe your race or ethnic background?

- ❍ White (1)
- ❍ Black or African American (2)
- ❍ Hispanic or Latino (3)
- ❍ American Indian (4)
- ❍ Alaskan Native (5)
- ❍ Asian (6)
- ❍ Other or multi-racial (7)
- ❍ Prefer not to answer (8)

Throughout this survey, "in a way not prescribed" includes any of the following:

- ***Used a product not prescribed to you Used for reasons other than as a treatment for pain***
- ***Used via an alternate route (e.g., snorted, injected, other routes not intended for the product***
- ***Used after tampering with the product (e.g., crushed)***
- ***Used in combination with alcohol, illicit drugs, or other prescription drugs without doctor approval***
- ***Used at a higher dose than prescribed***

6. In your lifetime, which of the following prescription opioid(s) have you used in a way not prescribed? Select all that apply.

- ❑ Buprenorphine (e.g., Subutex, Suboxone, Butrans) (1)
- ❑ Codeine (2)
- ❑ Fentanyl (e.g., Actiq, Duragesic, Fentora, Onsolis) (3)
- ❑ Hydrocodone ER (e.g., Zohydro ER, Hysingla ER) (4)
- ❑ Hydrocodone IR (e.g., Vicodin, Lortab) (5)
- ❑ Hydromorphone ER (e.g., Exalgo, Jurnista) (6)
- ❑ Hydromorphone IR (e.g., Dilaudid) (7)
- ❑ Meperidine (e.g., Demerol) (8)
- ❑ Methadone (9)
- ❑ Morphine ER (e.g., MS Contin, KADIAN, AVINZA, Oramorph SR, EMBEDA) (10)
- ❑ Morphine IR (e.g., MSIR) (11)
- ❑ Oxycodone ER (e.g., OxyContin, Xartemis XR) (12)
- ❑ Oxycodone IR non-combination product (e.g., Roxicodone, OxyIR) (13)
- ❑ Oxycodone IR combination product (e.g., Percocet, Tylox, Percodan, Combunox) (14)
- ❑ Oxymorphone ER (e.g., Opana ER) (15)
- ❑ Oxymorphone IR (e.g., Opana) (16)
- ❑ Tapentadol ER (e.g., Nucynta ER) (17)
- ❑ Tapentadol IR (e.g., Nucynta) (18)
- ❑ Tramadol ER (e.g., Ultram ER) (19)
- ❑ Tramadol IR (e.g., Ultram) (20)
- ❑ Other (Please specify) (21) ____________________
- ❑ I have never used a prescription opioid in a way not prescribed (22)

Display The following questions if tapentadol ER or IR were selected for Item 6:

7. What were your reasons for using prescription opioids in a way not prescribed? (Select all that apply)

- ❑ To provide better pain relief (1)
- ❑ To relax (2)
- ❑ To reduce my stress (3)
- ❑ To feel more outgoing (4)
- ❑ To feel more energetic (5)
- ❑ To feel high/buzzed/stoned (6)
- ❑ To experience psychedelic effects (e.g., hallucinations) (7)
- ❑ To enhance the recreational effects of other drugs/substances (8)
- ❑ To treat or prevent withdrawal symptoms (9)
- ❑ To ease the comedown from other drugs/substances (10)
- ❑ To treat emotional pain (11)
- ❑ To feel less depressed or anxious (12)
- ❑ Other reasons (Please specify) (13) ____________________

8. In your lifetime, which was the first prescription opioid that you used in a way not prescribed?

9. If you had to choose one, what is/was your preferred prescription opioid to use in way not prescribed?

10. If you had to choose one, what is/was your preferred route of administration when using prescription opioids in a way not prescribed?

- ❍ Swallow whole (1)
- ❍ Chew (2)
- ❍ Buccal (holding in cheek or sucking on it) (3)
- ❍ Parachute (rolled powdered or crushed tablet in toilet paper to ingest) (4)
- ❍ Drink in solution (5)
- ❍ Snort (6)
- ❍ Smoke (7)
- ❍ Inject (8)
- ❍ Rectal (plugging) (9)
- ❍ Sublingual (under tongue) (10)
- ❍ Transdermal (patch on skin) (11)
- ❍ Suck on transdermal patch (12)
- ❍ Chew on transdermal patch (13)
- ❍ Other (Please specify) (14) ____________________

11. If you had to choose one, what is/was your preferred drug/substance?

- ❍ Alcohol (1)
- ❍ Bath salts/MDPV (2)
- ❍ Benzodiazepines (e.g., Klonopin, Xanax) (3)
- ❍ Cocaine (4)
- ❍ Dissociative drugs (e.g., PCP, ketamine) (5)
- ❍ Hallucinogens/psychedelics (e.g., LSD, mushrooms) (6)
- ❍ Heroin (7)
- ❍ Inhalants (8)
- ❍ Marijuana/cannabis (9)
- ❍ MDMA or other emphathogenic drugs (10)
- ❍ Methamphetamine (11)
- ❍ Prescription antidepressants (e.g., Zoloft, Prozac) (12)
- ❍ Prescription opioids (e.g., OxyContin, Percocet, Vicodin) (13)
- ❍ Prescription stimulants (e.g., Adderall, Ritalin) (14)
- ❍ Other (Please specify) (15) ____________________

12. Do you still use prescription opioids in a way not prescribed?

- ❍ Yes (1)
- ❍ No (2)

13. If no, At what age did you stop using prescription opioids in a way not prescribed?
(list ages for selection of one age)

14. At what age did you first use Nucynta ER/IR in a way not prescribed?
(list ages for selection of one age)

15. Why have you used Nucynta ER/IR in a way not prescribed? (Select all that apply)

- ❑ To provide better pain relief (1)
- ❑ To relax (2)
- ❑ To reduce my stress (3)
- ❑ To feel more outgoing (4)
- ❑ To feel more energetic (5)
- ❑ To feel high/buzzed/stoned (6)
- ❑ To experience psychedelic effects (e.g., hallucinations) (7)
- ❑ To enhance the recreational effects of other drugs/substances (8)
- ❑ To treat or prevent withdrawal symptoms (9)
- ❑ To ease the comedown from other drugs/substances (10)
- ❑ To treat emotional pain (11)
- ❑ To feel less depressed or anxious (12)
- ❑ Other reason (Please specify) (13) ____________________

16, In your lifetime, which routes of administration have you used with Nucynta ER/IR? (Select all that apply)

- ❑ Swallow whole (1)
- ❑ Chew (2)
- ❑ Buccal (holding in cheek or sucking on it) (3)
- ❑ Parachute (rolled powered or crushed tablet in toilet paper to ingest) (4)
- ❑ Drink in solution (5)
- ❑ Snort (6)
- ❑ Smoke (7)
- ❑ Inject (8)
- ❑ Rectal (plugging) (9)
- ❑ Sublingual (under tongue) (10)
- ❑ Other (Please specify) (11) ____________________

A17. If you had to choose one, which route of administration do you prefer to use with Nucynta ER/IR?

A19. What methods have you used to manipulate or tamper with Nucynta ER? (Select all that apply)

- ❑ I have not manipulated the product prior to use (1)
- ❑ Chew (2)
- ❑ Break into smaller pieces (3)
- ❑ Crush/Grind/Shave (4)
- ❑ Dissolve/Soak (5)
- ❑ Filter dissolved product in liquid using cotton ball, coffee filter, or other material (6)
- ❑ Filter dissolved product in liquid using wheel filter, micron filter, or other type of filter (7)
- ❑ Heat (8)
- ❑ Cool/Freeze (9)
- ❑ Other (Please specify) (10) ____________________

18. Have you ever used Nucynta ER/IR in combination with other drugs or substances (e.g., alcohol, other drugs)?

- ❍ Yes (1)
- ❍ No (2)

19. What other drugs or substances have you used in combination with Nucynta ER/IR? (Select all that apply):

- ❑ Alcohol (1)
- ❑ Bath salts/MDPV (2)
- ❑ Benzodiazepines (e.g., Klonopin, Xanax) (3)
- ❑ Cocaine (4)
- ❑ Dissociative drugs (e.g., PCP, ketamine) (5)
- ❑ Hallucinogens/psychedelics (e.g., LSD, mushrooms) (6)
- ❑ Heroin (7)
- ❑ Inhalants (8)
- ❑ Marijuana/cannabis (9)
- ❑ MDMA or other emphathogenic drugs (10)
- ❑ Methamphetamine (11)
- ❑ Prescription antidepressants (e.g., Zoloft, Prozac) (12)
- ❑ Prescription opioids (e.g., OxyContin, Percocet, Vicodin) (13)
- ❑ Prescription stimulants (e.g., Adderall, Ritalin) (14)
- ❑ Other (Please specify) (15) ____________________

20. Where did you obtain Nucynta ER/IR? (Select all that apply)

- ❑ My own prescription from one doctor (1)
- ❑ My own prescription from multiple doctors (2)
- ❑ Given to me by a family member, friend, or acquaintance (3)
- ❑ Bought from a family member, friend, or acquaintance (4)
- ❑ Bought from a dealer (someone known to sell drugs) (5)
- ❑ Stolen (6)
- ❑ Internet sources (7)
- ❑ Other (Please specify) (8) ____________________

21. Where did you most often obtain Nucynta ER/IR?

22. What strength of tablets (in milligrams) of Nucynta ER have you used? (Select all that apply)

- ❑ 50 mg (1)
- ❑ 100 mg (2)
- ❑ 150 mg (3)
- ❑ 200 mg (4)
- ❑ 250 mg (5)
- ❑ Not sure/Do not know (6)

22A. What strength of tablets (in milligrams) of Nucynta IR have you used? (Select all that apply)

- ❑ 50 mg (1)
- ❑ 75 mg (2)
- ❑ 100 mg (3)
- ❑ Not sure/Do not know (4)

23. What is the highest dosage (in milligrams) of Nucynta ER/IR that you have consumed during one use session?

- ❍ Less than 100 mg (1)
- ❍ 100 - 250 mg (2)
- ❍ 251 - 500 mg (3)
- ❍ 501 - 750 mg (4)
- ❍ 751 - 1,000 mg (5)
- ❍ Greater than 1,000 mg (6)
- ❍ Not sure/Do not know (7)

24. Do you use different routes of administration with Nucynta ER/IR depending on the tablet strength that you have available?

- ❍ Yes (1)
- ❍ No (2)

25. Which route(s) of administration have you used with the 50 mg Nucynta ER/IR tablets?

25A. Which route(s) of administration have you used with the 75 mg Nucynta IR tablets?

26. Which route(s) of administration have you used with the 100 mg Nucynta ER/IR tablets?

27. Which route(s) of administration have you used with the 150 mg Nucynta ER tablets?

28. Which route(s) of administration have you used with the 200 mg Nucynta ER tablets?

29. Which route(s) of administration have you used with the 250 mg Nucynta ER tablets?

30. Do you still use Nucynta ER/IR?

- ❍ Yes (1)
- ❍ No (2)

31. How frequent is your use of Nucynta ER/IR?

- ❍ Daily (1)
- ❍ A few times a week (2)
- ❍ A few times a month (3)
- ❍ Every few months (4)
- ❍ I have only used it once or twice (5)

32. Why do you continue to use Nucynta ER/IR? (Select all that apply

- ❑ I have access to the drug (1)
- ❑ I enjoy how the drug makes me feel (e.g., the buzz, the nod) (2)
- ❑ I enjoy the psychedelic effects of the drug (e.g., hallucinations) (3)
- ❑ I have not experienced any negative side effects (4)
- ❑ I am not worried that I will experience negative side effects (5)
- ❑ The drug is inexpensive (6)
- ❑ I do not have better options available to me (7)
- ❑ The drug is effective at relieving my pain (8)
- ❑ The drug provides an effective high (9)
- ❑ It is easy for me to manipulate the product to use via my preferred route of administration (10)
- ❑ There is no (or less) stigma surrounding this drug compared to other drugs (11)

33. At what age did you stop your use of Nucynta ER/IR?

(list ages for selection of one age)

34. How frequent was your use of Nucynta ER/IR?

- ❍ Daily (1)
- ❍ A few times a week (2)
- ❍ A few times a month (3)
- ❍ Every few months (4)
- ❍ I only used them once or twice (5)

35. Why did you stop using Nucynta ER/IR? (Select all that apply)

- ❑ I do not have access to the drug (1)
- ❑ I do not enjoy how the drug makes me feel (e.g., the buzz, the nod) (2)
- ❑ I do not enjoy the psychedelic effects of the drug (e.g., hallucinations) (3)
- ❑ I have experienced negative side effects (4)
- ❑ I am worried that I will experience negative side effects (5)
- ❑ The drug is expensive (6)
- ❑ I have better options available to me (7)
- ❑ The drug is not effective at relieving my pain (8)
- ❑ The drug does not provide an effective high (9)
- ❑ It is difficult for me to manipulate the product to use via my preferred route of administration (10)
- ❑ There is more stigma surrounding this drug compared to other drugs (11)

36. Please rate each of the drugs listed below for how desirable/attractive they are to use in a way not as prescribed.

A rating of 0 means a drug is not at all desirable/attractive for non-medical use and a rating of 100 means a drug is the best drug imaginable to use non-medically.

As a reminder, "in a way not prescribed" includes any of the following:

- ***Used a product not prescribed to you Used for reasons other than as a treatment for pain***
- ***Used via an alternate route (e.g., snorted, injected, other routes not intended for the product***
- ***Used after tampering with the product (e.g., crushed)***
- ***Used in combination with alcohol, illicit drugs, or other prescription drugs without doctor approval***
- ***Used at a higher dose than prescribed***

* Thank you for completing this survey! Would you like to be considered for a follow-up interactive online chat with Inflexxion researchers regarding your use of tapentadol products? This chat will be completely anonymous.

❍ Yes (1)
❍ No (2)

* In order to be contacted with further information about the online chat, please provide either an email address (this email address does not have to include any personal information about you) or your Bluelight user name. A member of the Inflexxion research team will contact you shortly.

❍ Email address (1) ____________________
❍ Bluelight user name (2) ____________________
